# Multidimensional Nanocomposites of Epoxy Reinforced with 1D and 2D Carbon Nanostructures for Improve Fracture Resistance

**DOI:** 10.3390/polym10030281

**Published:** 2018-03-08

**Authors:** Juventino López-Barroso, Ana Laura Martínez-Hernández, José Luis Rivera-Armenta, Carlos Velasco-Santos

**Affiliations:** 1 Tecnológico Nacional de México/Instituto Tecnológico de Ciudad Madero, División de Estudios de Posgrado e Investigación, Centro de Investigación en Petroquímica, Pról. Bahía De Aldair y Ave. de las Bahías, Parque de la Pequeña y Mediana Industria, 89600 Altamira, Tamaulipas, Mexico; barrozo69@gmail.com; 2 Tecnológico Nacional de México/Instituto Tecnológico de Querétaro, División de Estudios de Posgrado e Investigación, Av. Tecnológico s/n, esq. Gral. Mariano Escobedo, Col. Centro Histórico, 76000 Santiago de Querétaro, Querétaro, Mexico; almh72@gmail.com (A.L.M.-H.); cylaura@gmail.com (C.V.-S.)

**Keywords:** carbon nanomaterials, graphene, carbon nanotubes, Izod fracture, multidimension composites

## Abstract

A hybrid nanocomposites based on epoxy reinforced with a combination of 1D and 2D carbon nanomaterials for improving impact resistance are reported. Multi-walled carbon nanotubes and oxidized-multi-walled carbon nanotubes are used as 1D nanoreinforcements, and graphene derivative materials such as graphene oxide and reduced graphene oxide are utilized as 2D nanoreinforcements. In this research, the impact resistance of epoxy matrix reinforced with 1D or 2D and the mixture of both nanomaterials is studied. The research is focused on evaluation of the influence of adding different combinations of nanomaterials into epoxy resin and their Izod impact response. Moreover, fracture surface of nanocomposites is observed by scanning electron microscopy. Images show differences between the surfaces of brittle nature on thermoset epoxy polymer and tough nanocomposites. Synergy created with 1D and 2D nanomaterials produces stable dispersions in the processing, reflected in the interface. The interactions in nanocomposites are evidenced by infrared spectra, principally on the peaks related to oxygenated functional groups present in nanomaterials and absent in polymer matrix. Consequently, an increase of 138% in fracture strength of nanocomposites is exhibited, in comparison to the neat epoxy matrix. In addition, hybrid nanocomposites were synthesized in two different methods to evaluate the influence of manufacturing method on final properties of nanocomposites.

## 1. Introduction

Epoxy resins have been used as a matrix in different high-performance composite applications, particularly in aerospace, aeronautics, automobile industry, military and sports equipment, as well as electronics and structural applications [1,2,3,4,5,6,7,8,9,10,11,12]. However, epoxy has some restrictions as a result of their crosslinked nature. This covalent network structure is reflected in mechanical properties such as low toughness, due to weak restriction on initiation and propagation of fracture; thus, the development of composites with high performance and multi-functionalities have been the topic of intensive research work [13]. 

The low toughness of epoxy resins has been enhanced with the use of carbon fibers in composites since the middle of last century, intensifying its aeronautics applications until now. Notwithstanding, to satisfy the needs for high demand on actual industrial applications, materials with high performance are required. Thus, novel materials have been developed with this focus, for instance, the multiscale composites. In these materials, the interfaces are enriched by the synergistic effect between a typical microreinforcement and nanostructure, creating better interfaces and consequently interesting improvements in mechanical properties as well as enhanced chemical and environment resistance [14,15]. However, challenges such as dispersion or impregnation of nanoreinforcement over fibers and the high volume of microreinforcement occupied have not been solved entirely [16].

On the other hand, composites reinforced with nanometric materials or “nanocomposites” have attracted attention due to the properties gained with a minimum amount of nanoreinforcements [17,18]. The performance of nanocomposites is related to their great surface area available, allowing a strong interface with polymeric matrices [19]. For instance, carbon allotropes, such as fullerenes, carbon nanotubes (CNT) and graphenic materials as graphene oxide (GO) or reduced graphene oxide (RGO), have been extensively studied on epoxy matrix due to interesting properties obtained with low concentrations of nanomaterials [1,5,20,21,22]. However, in these kinds of materials, the dispersion has not been solved completely, and it is needed to improve. 

The synergistic effect observed in the multiscale nanocomposites resulted in outstanding properties. Consequently, some interesting approaches have been developed through blends of two or more nanostructures to find the synergistic effect [8,11,14,23,24,25,26,27,28]. Their combination has resulted in hybrid nanocomposites with greater properties than nanocomposites with only one kind of nanoreinforcement, in agreement with Szeluga‘s definition. For example, the combination of metallic particles and carbon nanostructures such as Fe_3_O_4_–MWCNT improve the micro-cracks resistance of epoxy at cryogenic temperatures by orienting the nanoreinforcements with a magnetic field [14]. Other notable results were obtained by Wang et al. [24], who used carbon fiber reinforcement composite to achieve extraordinary mechanical improvements as a result of “a ferocious synergy” of silver nanoparticles and GO located on the interface of epoxy and carbon fiber. One of the most important parameters considered in nanomaterial for use as reinforcement is the dimension. Domun et al. gave relevant evidence of the influence of dimension when synthesized hybrid boron–carbon nanocomposites interchanging the dimension of 1D and 2D nanostructures used as reinforcements [23]. The fact that carbon allotropes display different geometries can help understand the dimensional classification related to electron transport [29] and the types of chemical bonds [30] for carbon materials.

Four classes of nanomaterials have been defined according to their dimension: Fullerenes as zero-dimensional (0D); CNT as unidimensional (1D); graphene and its derivatives as bi-dimensional (2D) (only if its thickness is below 100 nm); and 3D materials built from fewer dimensional nanostructures or their internal structure is in nanoscale [29]. Specifically, MWCNT are cylindrical graphene layers separated by a distance similar to graphite sheets [31], with length in micrometers, but a diameter on the nanoscale. 2D carbon nanomaterials are based on a single graphene sheet with an sp^2^ atomic arrangement, packed in honeycomb crystal lattice alike in CNT, but in the case of GO and RGO with the presence of oxygenated functional groups attached to their surfaces. Both 1D and 2D carbon nanomaterials have high aspect ratio. Consequently, superior mechanical properties are exhibited; in addition, high thermal and electrical conductivity derivate from electronic structure have been demonstrated [6,19,32]. However, each nanomaterial depending on dimension and shape could give not only different contribution at interface level with the polymeric matrix as reports mentioned for carbon–carbon hybrid nanostructures [8,11,27] but also to other fields of science, for example, drug delivery and disease therapy [33].

On the other hand, it is worth noting that functional groups on carbon nanomaterials surface of 1D or 2D materials also have played a decisive role in dispersion into the matrix due to oxygenated functional groups. Hence, mechanical properties of nanocomposites benefit from the presence on these pendant groups. For example, the functionalization of multi-walled carbon nanotubes (MWCNT) by ultrasonic waves in acids mixture method demonstrated an accurate amorphous carbon removal and the formation of oxidized functions, according to Wu et al. [25] and Tang et al. [34], favoring the interaction among 1D reinforcement and the polymeric matrix; even a chemical bonding could happen. Therefore, the nanocomposite materials in this research, used as reinforcements of epoxy matrix, include blends of MWCNT and their oxidized counterpart oxidized-multi-walled carbon nanotubes (O-MWCNT) as 1D reinforcement and graphene derivatives such as GO and RGO as 2D reinforcements. Consequently, the properties of hybrid multidimensional composites developed are discussed to elucidate the contribution of the dimension and the functionalization of carbon nanostructures from the impact resistance, which is a crucial property in aeronautic applications. The analysis of patterns created in the fracture zone of composites was a useful tool to the understanding of the interfaces created under the influence of the 1D and 2D nanomaterials and their blends. Moreover, the two methods used to synthesize the nanocomposites exhibited the notable differences when a direct synergy is created inside of polymer by the 3D carbon nanostructures instead of indirect synergy when reinforcements are added sequentially in layers of nanocomposites.

## 2. Materials and Methods

### 2.1. Materials and Equipment

Some reagents were supplied from Sigma-Aldrich and J. T. Baker and used as a received such as sulfuric acid (H_2_SO_4_, 98%, J. T. Baker, Ecatepec, Mexico), potassium permanganate (KMnO_4_, 99%, Sigma, St. Louis, MI, USA), chlorohydric acid (HCl, 37%, J. T. Baker), nitric acid (HNO_3_, 70%, J. T. Baker), Bisphenol-A di-glycidyl ether (DGEBA, Sigma, M.W. 340.41 g/mol) and ethylenediamine (EDA, Sigma, >99.5%, M.W. 60.1 g/mol). In addition, both ascorbic acid (LAA, 20 mM, J. T. Baker) and hydrogen peroxide (H_2_O_2_, 30%, J. T. Baker) were purchased from Baker. Carbon materials as MWCNT were supplied by SUNNANO (Sun Nanotek, Jiangxi, China, purity > 80%, with outer diameter 10–30 nm and length 1–10 µm) and graphite powder (GRA) (spectra-grade, ash content < 2 ppm) were purchased from Electron Microscopy Science (Hatfield, PA, USA).

### 2.2. Graphite Oxide Synthesis

2D materials such as GO and RGO were synthesized according to previous reports [35,36]. First, Graphite powder (GRA) was oxidized in the presence of H_2_SO_4_ and KMnO_4_ during 3 h in magnetic stirring, and then graphite oxide (GRAO) slurry was washed with HCl and dried in an oven (model FE-291AD, Felisa, Jalisco, Mexico) at 65 °C for 12 h in an atmosphere of air.

### 2.3. Graphene Oxide Preparation

One hundred milligrams of GRAO were dispersed in 10 mL of distilled water and bath-sonicated at room temperature (Autoscience 10200B, Tianjin, China, 40 kHz) during 3 h, then GO dispersion was dried at 65 °C for 24 h in an oven (model FE-291AD, Felisa, Jalisco, Mexico) in an air atmosphere.

### 2.4. Reduced Graphene Oxide

The reduction of GO was conducted adding LAA as a chemical reducing agent to a GO solution (1 mg/mL) at 95 °C for 20 min in magnetic stirring [35]. Afterwards, the obtained RGO was washed to neutral pH and dried at 65 °C for 12 h in an oven (model FE-291AD) in an air atmosphere.

### 2.5. Functionalization of CNT

Covalent functionalization of MWCNT nanotubes was developed in two steps. First, according to reported [34], 100 mg of MWCNT were suspended in a reflux system by a 3:1 molar mixture of H_2_SO_4_ and HNO_3_. They were sonicated in an ultrasonic bath at room temperature (Autoscience 10200B) for 30 min, and then washed with distilled water until neutral pH. Furthermore, to enhance the oxidation of CNT in a cylindrical homemade polytetrafluoroethylene (PTFE) chamber, 100 mg of previously acid modified MWCNT were mixed with 10 mL of hydrogen peroxide. Afterwards, MWCNT were irradiated with microwaves (MW) in a furnace at 1200 W (Panasonic, NN ST 778S, Shanghai, China). This process lasted 8 min, with intermittent periods of 1 min of MW (1 min on, 1 min off) to avoid catastrophic damage to the 1D nanomaterial, considering the advice of de la Luz et al. [37]. Finally, the slurry obtained was washed until neutral pH and dried for 24 h at 60 °C.

### 2.6. Synthesis of Nanocomposites

For the synthesis of nanocomposites, DGEBA and EDA were used as a matrix and hardener, respectively. Nanoreinforcements were added to the matrix in different quantities (see Table 1). These percentages were selected based on different reports [1,20,38]. These quantities of reinforcements normally avoid agglomerations, but are sufficient concentrations to modify significantly the mechanical performance.

Two methods of transfer to mold the resin were employed. First, for multidimensional nanocomposites (MD), the process consisted of mixing for one h in an ultrasonic bath (40 kHz) 24 g of DGEBA and fit quantity of nanoreinforcements, and then hardener was aggregated followed by hand mixing for 60 s. This mixture was transferred to mold with geometry according to the American Society for Testing and Materials (ASTM) D246 norm and cured in an oven at 46 °C for 1 h. Second, four layers of 6 g of DGEBA with the right amount of nanoreinforcements (see Table 1) were deposited sequentially (the nanomaterials were previously dispersed for 1 h in an ultrasonic bath with frequency of 40 kHz). In other words, one layer was placed and cured at 46 °C for 1 h, and, once the material was cured, a new layer was placed. This procedure was repeated four times. In these multilayer-multidimensional nanocomposites (ML), the four layers were intercalated with one layer of 2D reinforcement followed by 1D reinforcement.

### 2.7. Characterization of Carbon Nanotubes

Fourier Transforms Infrared (FTIR) and Raman spectra of carbon nanoreinforcements were recorded using Bruker Tensor 37 (Bruker, Billerica, MA, USA) in transmittance mode with ATR accessory at 32 scans with a resolution of 1 cm^−1^ and Bruker Senterra (Bruker) with a laser of 785 nm with 4 cm^−1^ of resolution, respectively, in both cases the data obtained were processed by Origin Lab 9.0 software. Moreover, transmission electron micrographs of nanostructures were performed with JEOL JEM-1010 (JEOL, Peabody, MA, USA) with a voltage of operation at 80 kV.

### 2.8. Characterization of Nanocomposites

Infrared spectra of nanocomposites were obtained by Bruker Tensor 37 IR equipment (Bruker) with ATR accessory, at 32 scans. Izod impact tests were conducted in a Tinius Olsen 503 A model pendulum (Tinius Olsen, Horsham, PA, USA), at room temperature. The Izod specimens (64 × 12.7 × 3.2 mm^3^) were notched at the center of sample by the Tinius Olsen 899 specimen notcher (Tinius Olsen) designed to avoid damaging specimens, at room temperature according to the ASTM D256 norm. The hammer of the impact test machine had a capacity of 2.78 J, and arm distance of 0.61 m. A minimum of five replicates were used for all mechanical testing. For both cases, infrared spectra of nanocomposites and impact test results, the data obtained were processed by Origin Lab 9.0 software. The micrographs of scanning electron microscopy (SEM) were obtained using HITACHI TM-1000 equipment (Hitachi, Tokyo, Japan) through backscattered electrons in a low vacuum at 15 kV.

## 3. Results and Discussion

### 3.1. Infrared and Raman Spectroscopies of 1D and 2D Nanostructures

**Figure 1:**
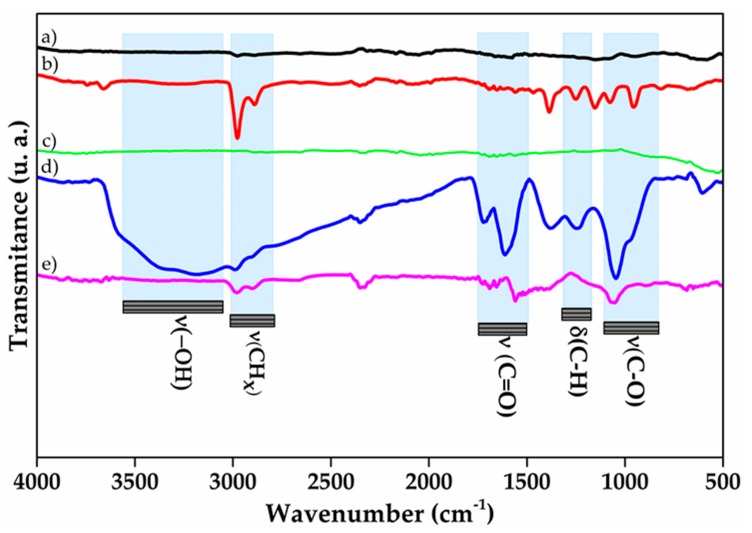
Infrared spectrum of nanocarbon forms: (**a**) pristine MWCNT (multi-walled carbon nanotubes); (**b**) O-MWCNT (oxidized multi-walled carbon nanotubes); (**c**) GRA (graphite); (**d**) GO (graphene oxide); and (**e**) RGO (reduced graphene oxide).

Figure 1 shows the spectra of carbon nanomaterials. The IR spectrum for pristine MWNTC remains without signals. On the other hand, the O-MWCNT spectrum shows characteristic signals of different oxygenated functional groups, for instance at 3280 cm^−1^ which is attributed to *ν*(–OH) of hydroxyl groups [32]. Additionally, characteristic signals of both carboxylic acids *ν*(C=O) at 1693 cm^−1^ and carboxylate *ν*(C=O) at 1653 cm^−1^ and 1557 cm^−1^ are observed [39]. Moreover, signals at 1250 cm^−1^, 1153 cm^−1^, and 1075 cm^−1^ correspond to *ν*(C-O) from ethers formed during the oxidation [40]. Peaks at 2977 cm^−1^ and 2980 cm^−1^ correspond to *ν*(C-H) of CH_x_ groups forming as defects in walls of MWCNT. In addition, their corresponding *δ*(C-H) mode is observed at 1467 cm^−1^ and 1385 cm^−1^ [41].

Similar to pristine MWCNT, the GRA spectrum does not show any peaks (Figure 1). However, after the oxidation, a broad peak appears at ≈3183 cm^−1^ that can be attributable to *ν*(–OH) of hydroxyl groups. Signals at 1716 cm^−1^ and 1378 cm^−1^ show activity for *ν*(C=O) of carboxylic acids and carboxylates, respectively [42]. Additionally, the peaks at 1249 cm^−1^ and 1047 cm^−1^ can be assigned to the activity of oxirane rings formed on the surface of GO [17]. All the signals that appear in GO point to a successful formation and bonding of oxygenated functional groups over graphitic layers. Nonetheless, for RGO IR spectrum, there exists a diminishing in oxygen functionalities bands. This difference on bands can be explained as the removal of oxygenated moieties from graphitic layers after the reduction with L-AA, and therefore only two smooth peaks at 1700 cm^−1^ and 1051 cm^−1^ can be observed due to remaining groups such as carbonyls and alkoxy [43], respectively.

**Figure 2:**
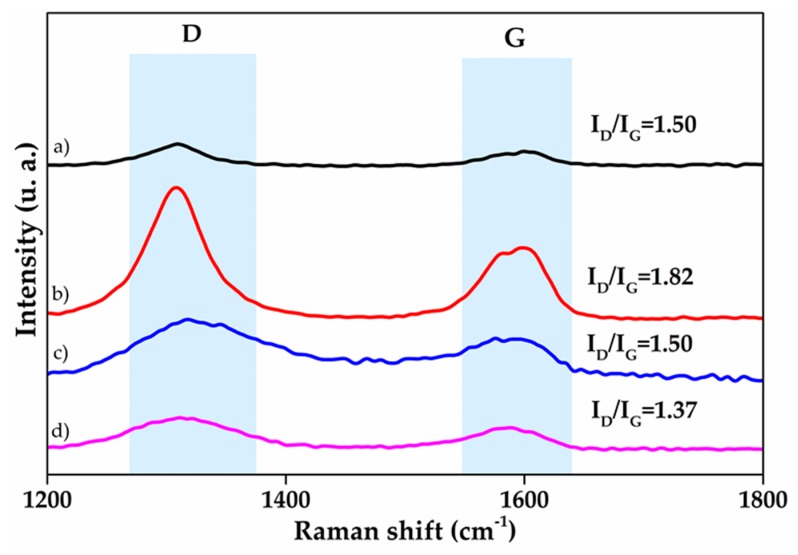
Raman signals of: (**a**) MWCNT; (**b**) O-MWCNT; (**c**) GO; and (**d**) RGO.

Raman spectra of carbon materials are shown in Figure 2. In CNT spectra, characteristic signals are observed at 1309 cm^−1^ and 1599 cm^−1^ related to D and G bands. The first one is activated in the first order scattering process of sp^2^ carbon nanostructures [44], and it is attributed to defects on the walls or disorder in graphitic materials [28,31], which disrupt or diminish the symmetry in the characteristic hexagonal arrangement in carbon allotropes with sp^2^ hybridization. G band at approximately 1599 cm^−1^ has been related to the presence of the sp^2^ phase of high crystalline graphitic structures (graphitic lattice mode E_2g_), the peak corresponds to tangential in-plane bond stretching of the sp^2^ C–C atoms in hexagonal ring [45,46,47].

Neither D band nor G band of O-MWCNT shows an important shift in comparison with the spectrum of pristine MWCNT. However, the intensity ratio (I_D_/I_G_) is considered important parameter to quantify disorder in carbon nanostructures [48]. I_D_/I_G_ ratio shows significant increases from 1.5 to 1.82 for the values of MWCNT and O-MWCNT, respectively. This is related to an increase of defects on walls of MWCNT after the oxidation with ultrasound and microwaves, and it confirms the superficial modification of 1D nanostructures with oxygenated moieties, as IR spectrum shown. 

For the Raman spectra of 2D nanostructures, the characteristics signals corresponding to carbon nanomaterials are shown in Figure 2: D band from the electronic and geometrical structure through the double resonance process, and G signal from all sp^2^ carbon allotropes [48]. In GO spectrum, the signals (D and G) appear around 1318 cm^−1^ and 1575 cm^−1^, respectively. After the reduction through LAA, D band suffers a red shift to 1308 cm^−1^ and becomes narrow in comparison to GO spectrum. This displacement can be attributed to the restoration of sp^2^ phase in RGO. On the other hand, G band shows a blue shift (1588 cm^−1^) as a result of the creation of small crystallites of sp^2^ domains, which its boundaries consider as defects in Raman measurements [35]. The sp^2^ restoration can be corroborated by the I_D_/I_G_ ratio decrease, from 1.5 to 1.37, which can be interpreted as a tendency toward I_D_/I_G_ graphite ratio (0.032) [49], consequently confirming the reduction of GO by removal oxygenated functional groups, also in agreement with infrared results.

### 3.2. Transmission Electron Microscopy of Carbon Nanostructures

**Figure 3:**
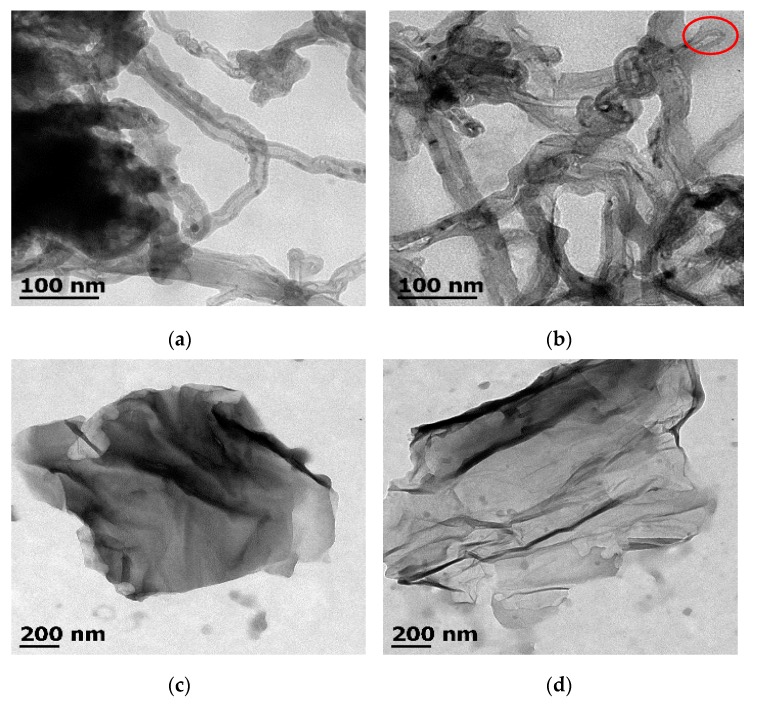
TEM micrographs of: (**a**) pristine MWCNT; (**b**) O-MWCNT; (**c**) GO; and (**d**) RGO.

Figure 3 illustrates the images of both 1D and 2D reinforcements observed by transmission electron microscopy (TEM) before and after the modifications. It is possible to observe different changes in their structure and morphology. Figure 3a shows intricate, tubular and hollow structures with diameters around of ≈1040 nm and length of a few microns, corresponding to as-received MWCNT. MWCNT are densely agglomerated, and the presence of amorphous carbon is over most of them. However, after functionalization, O-MWCNT shows walls without carbonaceous deposits, and, in a few cases, tips are open, as shown in Figure 3b in a red circle, which is an indicator of a successful functionalization without catastrophic damage in aspect ratio and walls of O-MWCNT. Thus, it is shown that ultrasonic and microwave energy in accurate conditions not only cleans the nanotube walls but also induces the formation of oxygenated moieties which are more compatible with matrix instead of the inert surface in pristine MWCNT [34,37,50].

On the other hand, there are no significant changes in morphology for 2D materials such as GO (Figure 3c) and RGO (Figure 3c). TEM images show a flaky shape, wrinkled, smooth sheet-like structures [51] with large dimensions (≈1.2 µm × 1.5 µm) for both materials. This fact allows deducing that the whole sheets are not broken after oxidation; they are modified on the surface (as corroborated by other techniques).

### 3.3. Infrared Spectroscopy of Nanocomposites

**Figure 4:**
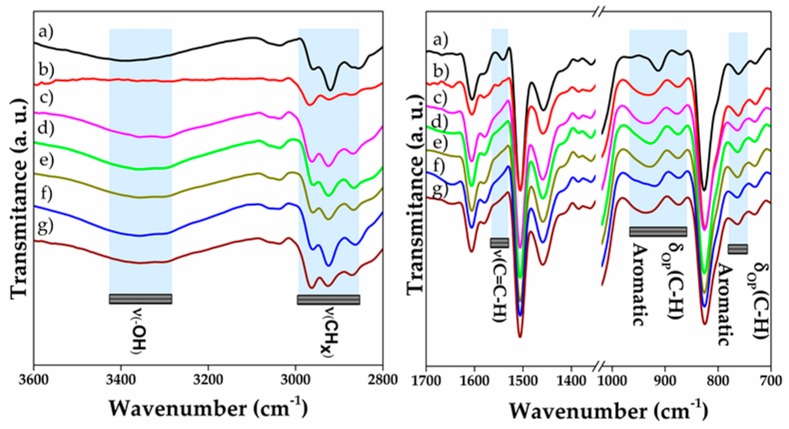
Infrared spectra of multidimensional nanocomposites of: (**a**) Neat epoxy MD; (**b**) MWCNT 0.1 wt % MD; (**c**) O-MWCNT 0.1 wt % MD; (**d**) GO 0.1 wt % MD; (**e**) RGO 0.1 wt % MD; (**f**) MWCNT-RGO 0.1–0.1 wt % MD; and (**g**) O-MWCNT-GO 0.1–0.1 wt % MD.

Figure 4 illustrates infrared spectra of multidimensional nanocomposites. The bands related to neat epoxy predominates in nanocomposite spectra due to the low amount of nanomaterials aggregated. Nevertheless, some differences are clearly observed because of the polymer–reinforcement interactions. A typical signal of *ν*(–OH) is seen in ≈3389 cm^−1^ for neat epoxy MD and for nanocomposites spectra the band shows a shift to lower wavenumbers (≈3358–3353 cm^−1^). This can be attributed to the rise in the number of hydrogen bonds or dipole–dipole interactions as consequence of the increment of –OH groups produced in epoxide ring opening reaction as well as presence of nanoreinforcements [52]. In addition, the CH_x_ region shows an important modification, for instance, *ν*_s_(CH_2_) signal in nanocomposites reinforced with nanostructures with high oxygenated moieties is strongly influenced. For neat epoxy MD, this signal is observed at ≈2856 cm^−1^, in contrast to signal for O-MWCNT 0.1 wt % MD and GO 0.1 wt % MD, the peaks of which are shown at 2868 cm^−1^ and 2867 cm^−1^, respectively. In addition, the signal of *ν*_s_(CH_2_) from O-MWCNT-GO 0.1–0.1 wt % MD appears at ≈2871 cm^−1^, indicating the strong influence of oxygenated moieties over aliphatic chains of polymer [53]. Finally, the signals at 912 cm^−1^, 870 cm^−1^, and 761 cm^−1^ are attributed to *δ*_out-of-plane_(C–H) present not only in aliphatic chains but also in aromatic domains of epoxy resin. These signals are shifted in composite spectra to higher wavenumber in comparison to neat epoxy MD; thus, it is suggested that hydrogen bond formation could promote restriction of the movement between chains and reinforcements, acting as one of the toughening mechanisms in nanocomposites, as mentioned previously by other authors [6].

### 3.4. Impact Properties

**Figure 5:**
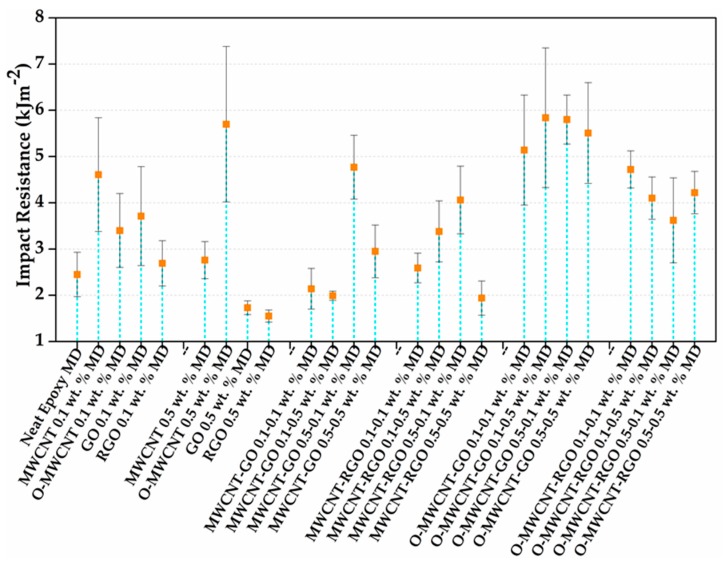
Izod impact resistance values for multidimensional nanocomposites.

**Figure 6:**
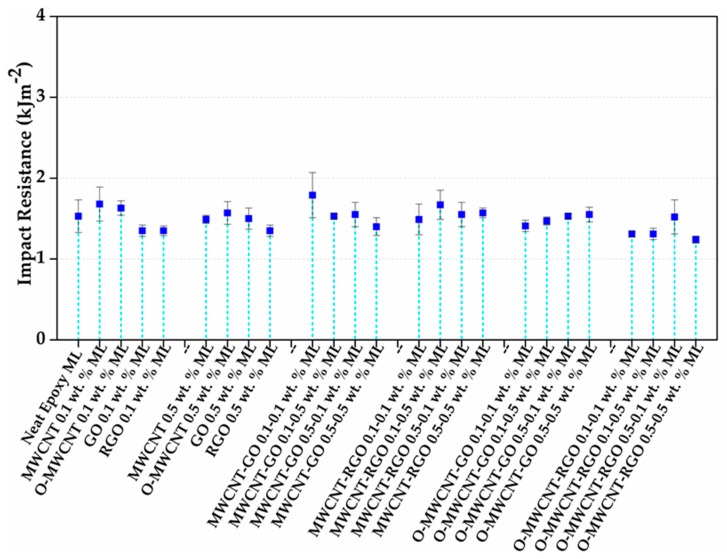
Izod impact resistance values for neat epoxy and multilayer nanocomposites.

The results of Izod impact test are reported regarding energy per unit of the cross-sectional area under the notch. Impact resistance results of nanocomposites in comparison with matrix are shown in Figure 5 for multidimensional nanocomposites, and in Figure 6 for multilayer nanocomposites.

In Figure 5, neat epoxy MD shows the impact resistance of 2.45 kJm^−2^; this value is notably increased to 4.61 kJm^−2^ (≈88% rise) in the nanocomposite with 0.1 wt % of MWCNT. At the same time, for the 1D oxidized nanoreinforcement (O-MWCNT 0.1 wt % MD), a value of 3.40 kJm^−2^ (≈37%) is obtained. This can be related to the nanotube aspect ratio, which is intact, in the case of MWCNT, in contrast of O-MWCNT, where aspect ratio could be diminished due to the acid treatments used in the functionalization. Nevertheless, when the quantity on O-MWCNT is increased to 0.5 wt % (O-MWCNT 0.5 wt % MD), the impact resistance is enhanced to 5.7 kJm^−2^ (≈132%); in comparison with MWCNT 0.5 wt % MD, this composite only shows a modest increase of approximately 13% (2.76 kJm^−2^). This can be explained in terms of better dispersion reached for O-MWCNT at 0.5 wt % (O-MWCNT 0.5 wt % MD) due to the functional groups produce an interfacial repulsion between 1D nanostructures [8]; consequently, agglomerates are broken down, and the web of 1D nanomaterials could be extended. Therefore, the interface created can be related not only to Van der Walls forces but also the possibility of covalent bonds could be formed during the crosslinking between matrix and reinforcement (which could be the main reason to add an extra quantity of EDA during the synthesis of O-MWCNT 0.5 wt % MD sample for a complete cure). However, in the composite containing 0.5 wt % of MWCNT (MWCNT 0.5 wt % MD), no improvement is obtained, indicating that nanotubes are agglomerated when the concentration is increased in the polymer [22].

In the composites reinforced with 0.1 wt % of 2D materials, the sample RGO 0.1 wt % MD exhibits an impact resistance of 2.69 kJm^−2^ that corresponds to a moderate rise of approximately 10% with respect to the neat epoxy MD. In contrast, composites GO 0.1 wt % MD reached a notable increase value of 3.71 kJm^−2^ in fracture strength, with a rise of ≈51% in comparison with the matrix. Thus, the results suggest both GO and RGO have a positive effect on impact resistance of epoxy. The GO achieved a higher increment on impact resistance than RGO. This can be explained by the following. First, dispersion could be improved by oxygenated moieties generating polar interactions, for instance, hydrogen bond and Coulombic interactions; and even covalent bonds probably could be formed, as others authors have mentioned [6,54,55]. Second, even though GO sheets possess disrupted sp^2^ structure, they maintain outstanding mechanical properties; additionally, their lateral flexibility and high aspect ratio promote notable improvements, if oxidized sheets are used as reinforcement [6]. In contrast, RGO cannot transfer its mechanical properties of the restored aromatic rings [8,56]; this sp^2^ nature recovery provokes more π–π interactions. Consequently, re-stacking of the RGO sheets happens, impacting on the available surface area and wettability of reinforcement. 

For nanocomposites GO 0.5 wt % MD and RGO 0.5 wt % MD, lower impact resistance values than the matrix (less 29% and 37%, respectively) are observed. These results can be linked to poor dispersion and excessive aggregation of the 2D nanocarbon structures [6], and are similar to those reported by Zhang et al. [20], who pointed out optimal improvements on impact resistance at 0.2 wt % of hyperbranched polyamine-ester functionalized GO added to epoxy; after this concentration, the impact strength of nanocomposites tends to decrease as a result poor dispersion and concentrated stress appeared.

In Figure 4, different blends of 1D and 2D are evaluated to investigate the synergetic effect produced by the carbon nanomaterials depending on dimension. Nanocomposites reinforced with O-MWCNT show outstanding properties in the four combinations realized (O-MWCNT-GO 0.1–0.1 wt % MD, O-MWCNT-GO 0.1–0.5 wt % MD, O-MWCNT-GO 0.5–0.1 wt % MD, and O-MWCNT-GO 0.5–0.5 wt % MD), because the impact properties suggested that the effect of functional groups in nanomaterials improves the mechanical properties of composites. Nevertheless, a real synergistic effect can only be attributable to the composites O-MWCNT-GO 0.1–0.5 wt % MD, MWCNT-GO 0.5–0.1 wt % MD, O-MWCNT-GO 0.1–0.1 wt % MD, O-MWCNT-RGO 0.1–0.1 wt % MD, MWCNT-RGO 0.5–0.1 wt % MD, and O-MWCNT-RGO 0.1–0.5 wt % MD. Hence, the impact strength of these MD nanocomposites in comparison with neat epoxy MD and unidimensional nanocomposites (either 1D or 2D) gives evidence of the formation of the synergistic effect. 

For instance, the composite O-MWCNT-GO 0.1–0.5 wt % MD with impact resistance of 5.84 kJm^−2^ show increments of 138% in comparison of neat epoxy MD, 72% in comparison to O-MWCNT 0.1 wt % MD and 111% in comparison of GO 0.5 wt % MD. Moreover, the nanocomposite MWCNT-GO 0.5–0.1 wt % MD, show a similar tendency, corroborating the synergistic effect previously mentioned. In this case, these values can be related to the fact that GO acts as dispersant agent for MWCNT. The improvements as a result of the synergetic effect of two nanostructures have also been observed on other properties, for example, tensile properties [8,28], Charpy impact properties [8], and electric and thermal behavior of the hybrid nanocomposites [57].

The notable increase in impact properties of the nanocomposites can be explained concerning different proposed mechanisms: First, for MWCNT, two processes about fracture responses in nanocomposites have been reported. Pull out and fracture deflection mechanisms are accepted, even though intermediate mechanisms influence them, such as “bridging” effect and the polymer–nanoreinforcements interface; this latter is enormously influenced by functionalization of 1D materials [2,8]. Second, for 2D materials, three failure modes have been reported in fracture response: (a) crack pinning; (b) separation of 2D graphitic layers; and (c) shear failure as consequence of differences within fracture surfaces [5]. Third, when 1D and 2D materials are combined, a combination of the mechanisms described previously has been found. That combination creates a synergistic effect reflected on final properties of the composite, as can be illustrated in results of Izod test in this work, principally for the family of the composites reinforced with O-MWCNT and GO. This mixed mechanism has been reported by other authors, where increments on flexural, hardness and impact properties of nanocomposites are attributed to the synergistic effect between MWCNT and nanodiamonds (ND) [8]. In addition, aromatic regions present in either 1D or 2D materials can promote π–π interactions making a stable dispersion through the “adsorption” or entrapment of 1D nanostructures by 2D materials, and also topological and reactive sites on GO would lead a better interlocking and consequently improvements on interface [10,27]. 

In the case of multilayer nanocomposites, fewer differences are observed between the combinations, especially for MWCNT-GO 0.1–0.1 wt % ML; this sample shows an increase of 17% in comparison to neat epoxy ML, 6.5% for MWCNT 0.1 wt % ML and 32.5% for GO 0.1 wt % ML. These modest modifications could be influenced by the synthesis method: in these nanocomposites, another interface between layers is acting as reducer on impact resistant and also the indirect synergy originated from the combination of 1D and 2D nanostructures is less effective than the direct interaction that can be observed in multidimensional nanocomposites (Figure 6).

### 3.5. Microstructure of 1D–2D Nanocomposites

**Figure 7:**
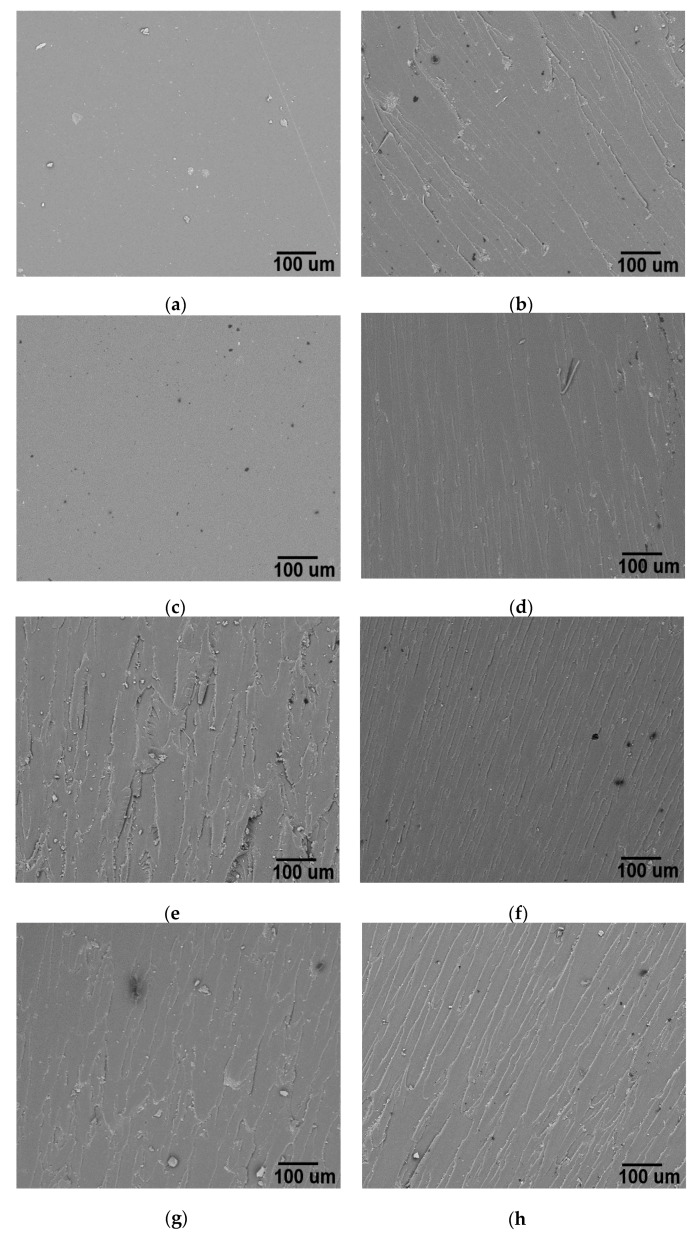
Micrograph of: (**a**) Neat epoxy MD (**b**) RGO 0.5 wt % MD; (**c**) MWCNT 0.1 wt % MD; (**d**) O-MWCNT 0.1 wt % MD; (**e**) RGO 0.1 wt % MD; (**f**) GO 0.1 wt % MD; (**g**) MWCNT-RGO 0.1–0.1 wt % MD; and (**h**) O-MWCNT-GO 0.1–0.1 wt % MD.

Figure 7 shows SEM images after impact tests of epoxy matrix and nanocomposites. Figure 7a shows the typical fracture surface of a brittle polymer, with low absorption of fracture energy and consequently poor toughness. The fracture surface of this kind of materials is smooth and mirror-like [7,58], which is directly related to the nature of high crosslink density epoxy systems and is considered a barrier to strain. However, in the samples containing carbon nanostructures, the epoxy fracture resistance increases considerably, as Izod results exhibit; therefore, remarkable changes in nanocomposites surface are observed.

Figure 7b–h shows different patterns of fracture surface in nanocomposites. According to the literature, typical U or V shapes and river-like patterns are formed when fracture front is deflected. In nanocomposites, this front of fracture is deviated by nanostructures, so more energy is required to propagate the fracture [3,7,9]. In addition, the length of river patterns indicates the degree of plastic strain to fracture [59]. These differences in fracture patterns are produced from the nanoparticle shape and size, interactions amongst nanostructures and the interface created with the matrix [60].

Figure 7c,d shows the fracture surface of nanocomposites reinforced with 1D materials. In Figure 7c, the surface of MWCNT 0.1 wt % MD nanocomposite exhibits a smooth surface of fracture comparable to neat epoxy MD, indicating a brittle fracture. Despite their brittle fracture, MWCNT 0.1 wt % MD nanocomposites show better mechanical properties than the epoxy matrix with a high impact strength as Izod results showed. This could be explained from the high aspect ratio in MWCNT which promotes certainly restriction of polymeric chains, attributable to mechanic interlocking between MWCNTS and epoxy but in some cases, their high aspect ratio can provoke the agglomeration of tubes, as it is mentioned in previous works [61]. On the other hand, fracture surface of O-MWCNT 0.1 wt % MD nanocomposites (Figure 7d) shows clear roughness and also V-shape and river-like patterns; these latter with length over 10 µm, related to a high plastic strain to fracture [59]. Impact resistance observed might well be originated for improvements in both dispersion and interface, as a result of functional groups attached to the MWCNT surfaces.

In the case of nanocomposites with a low percentage of 2D carbon nanomaterials, superior impact resistance to matrix is observed. In both cases, i.e., for nanocomposites GO 0.1 wt % MD and RGO 0.1 wt % MD, the fracture zone observed by SEM show intricate and deep-roughness surfaces. Nevertheless, the fracture surface of composite GO 0.1 wt % MD (Figure 7f) clearly has a superior tendency to be more tortuous than RGO 0.1 wt % MD nanocomposite (Figure 7e). This behavior indicates an indirect formation and a better, uniform dispersion in the sample containing GO [9]. This is in agreement with a previous report [62] where notorious changes on fracture surface for nanocomposites with different levels of dispersion are observed. Predominantly, fracture surface of highly dispersed 2D nanomaterials is characterized not only by a high number of irregular and fine river-like patterns but also long hackles and ribbons, as observed in Figure 7f. Notwithstanding, if 2D nanomaterials at high loads are aggregated. mechanical properties decreased abruptly despite the pattern shown in fracture zone (Figure 7b), contrasting with some reports which attributed better mechanical properties when a high load of 2D carbon nanomaterial is added [49]. 

The fracture surfaces of nanocomposites MWCNT-RGO 0.1–0.1 wt % MD and O-MWCNT-GO 0.1–0.1 wt % MD (Figure 7g,h) are a kind of average between their respective 1D–2D fracture surface materials. Particularly results more notorious in the case of O-MWCNT-GO 0.1–0.1 wt % MD nanocomposite (Figure 7h). In these materials, longer and deeper patterns than in MWCNT-RGO 0.1–0.1 wt % MD nanocomposite are observed. Thus, the influence of toughening mechanisms involving the synergetic effect of two different geometries can be comparable to the interaction of platelet-like (2D materials) and fiber-like (1D materials) structures with their respective contributions [63]. Other authors attribute the improvements to flexural, electrical and electrochemical properties [64,65]. It has been mentioned that these geometries form 3D hierarchical structures, modifying the fracture response [8,66], with a notable increase in the energy required to fracture the nanocomposite, as Izod test results show in this research. Moreover, in these SEM images, there are no visible agglomerates in the fracture zone, which could diminish mechanical properties. These results related to the surface on fracture zone confirm indirectly the synergistic effect created from intercalation of 1D–2D materials, as mentioned in some previous works [11,67,68]. However, this research evidences that this effect depends on many factors and can be enhanced if functionalization and accurate dispersion methods are used.

**Figure 8:**
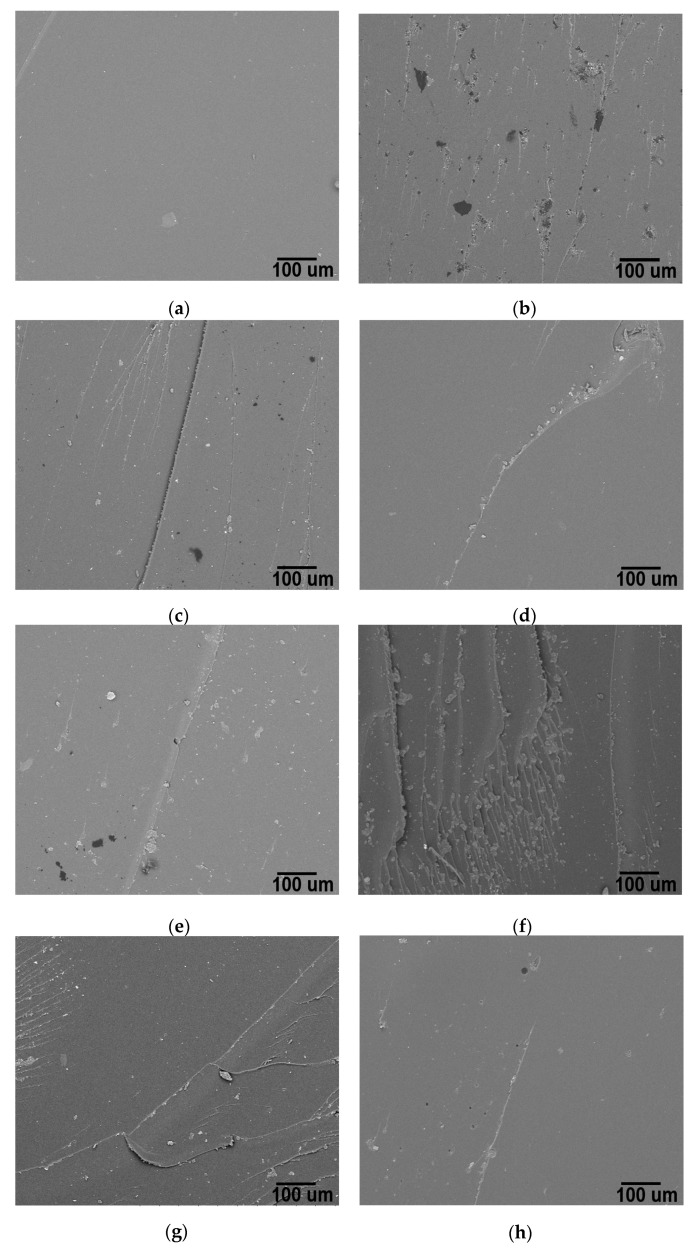
Micrograph of: (**a**) Neat epoxy ML (**b**) RGO 0.5 wt % ML; (**c**) MWCNT 0.1 wt % ML; (**d**) O-MWCNT 0.1 wt % ML; (**e**) RGO 0.1 wt % ML; (**f**) GO 0.1 wt % ML; (**g**) MWCNT-RGO 0.1–0.1 wt % ML; and (**h**) O-MWCNT-GO 0.1–0.1 wt % ML.

Figure 8 shows images of fracture zone of multilayer nanocomposites. Neat epoxy ML exhibits a smooth surface that indicates a brittle fracture, as in multidimensional nanocomposites [7,20,58]. Figure 8c–e,h corresponds to MWCNT 0.1 wt % ML, MWCNT 0.1 wt % ML, RGO 0.1 wt % ML and O-MWCNT-GO 0.1–0.1 wt % ML, respectively. In these images, a few river patterns are randomly distributed in the fracture zone. This surface could be attributed to the poor interaction between reinforcements and the matrix; this latter could be related to the manipulation of few quantity of resin and amine in the synthesis of ML composites in each layer. This fact agrees with mechanical results, inasmuch as, in the majority of these composites, modest improvements were observed, but also values below the impact strength of neat epoxy ML were registered. These results can be attributed to the route of synthesis, as previously mentioned. On the other hand, in Figure 8b,f,g, associated to RGO 0.5 wt % ML; GO 0.1 wt % ML and MWCNT-RGO 0.1–0.1 wt % ML nanocomposites, respectively, it is possible to observe some regions with U shape and river-like patterns in the fracture surface. These patterns features are normally related to better dispersion of nanoreinforcements among the matrix, indicating interactions between carbon materials and the epoxy matrix [69], however, in any composite, the impact resistance was above the value of the neat epoxy ML. These results could explain how the synthesis method plays an important role on the final results (Figure 5 and Figure 6), where the scatter on impact resistance values of the multidimensional nanocomposites (MD) is higher in comparison to multilayer nanocomposites values. The direct synergetic effect created in multidimensional nanocomposites (MD) produces an internal network which can vary as a result of different reinforcements–matrix and reinforcement–reinforcement interactions; this latter interaction is not possible in multilayer nanocomposites (ML)

## 4. Conclusions

The chemical modification developed in this study is successful, and minimum damage on MWCNT walls was observed. Spectroscopic techniques such as Infrared and Raman of MWCNT corroborated typical signals of 1D carbon nanostructures after oxidation, even when high energy treatments (ultrasonic/microwaves) are used. In addition, the typical morphology of MWCNT with minimum damage is undoubtedly observed on TEM. 

According to results in Izod test, the presence of functional groups on 2D materials such as GO can be beneficial to improve and stabilize their dispersion on polymeric matrix. RGO does not show remarkable improvements as reinforcement despite its restoration of sp^2^ bonds and the presence of remnant oxygenated moieties. However, the addition of O-MWCNT as reinforcements could avoid RGO stacking, as suggested by the results on impact test of O-MWCNT-OGR 0.1–0.1 wt % MD nanocomposite. Two important things can be inferred: the reduction of GO with LAA restores mechanical properties on graphitic layers, but the re-stacking is inevitable; and the addition of O-MWCNT result in a synergistic effect amongst 1D–2D nanomaterials that may prevent π–π re-stacking of RGO. Thus, the importance of functional groups on carbon nanostructures in the dispersion method is evident. In addition, the concentrations of reinforcements used in this research play an essential role in the mechanical properties of nanocomposites. 

The most outstanding impact resistance is acquired when both GO and O-MWCNT are aggregated to epoxy matrix. This clear tendency in O-MWCNT-GO mixtures for high values (up to ≈138% higher than neat epoxy) of impact resistance gives evidence for the relevance of functional groups in the stabilization of the dispersion. This was corroborated indirectly in SEM analysis by the differences in the roughness of surfaces as a result of modification of interfaces between matrix and reinforcements. In addition, homogenous patterns on fracture zone of these nanocomposites can give some signs of well-dispersed carbon nanostructures and their effect on the impact properties.

## Figures and Tables

**Table 1:** Nomenclature for Multidimensional (MD) and Multilayer-Multidimensional (ML) nanocomposites according to the quantities and type of added 1D and 2D nanoreinforcements.

Neat Epoxy					
MWCNT 0.1 wt %	MWCNT 0.5 wt %	MWCNT-GO 0.1–0.1 wt %	MWCNT-RGO 0.1–0.1 wt %	O-MWCNT-GO 0.1–0.1 wt %	O-MWCNT-RGO 0.1–0.1 wt %
O-MWNTC 0.1 wt %	O-MWNTC 0.5 wt %	MWCNT-GO 0.1–0.5 wt %	MWCNT-RGO 0.1–0.5 wt %	O-MWCNT-GO 0.1–0.5 wt %	O-MWCNT-RGO 0.1–0.5 wt %
GO 0.1 wt %	GO 0.5 wt %	MWCNT-GO 0.5–0.1 wt %	MWCNT-RGO 0.5–0.1 wt %	O-MWCNT-GO 0.5–0.1 wt %	O-MWCNT-RGO 0.5–0.1 wt %
RGO 0.1 wt %	RGO 0.5 wt %	MWCNT-GO 0.5–0.5 wt %	MWCNT-RGO 0.5–0.5 wt %	O-MWCNT-GO 0.5-0.5 wt %	O-MWCNT-RGO 0.5–0.5 wt %

Note: Nanocomposites marked with the legend “MD” correspond to multidimensional and legend “ML” corresponds to multilayer nanocomposites. Table 1 shows the percentages used in both transfer methods to synthesize the nanocomposites.
